# Clinical study on freehand of bicortical sacral screw fixation with the assistance of torque measurement device

**DOI:** 10.1186/s12891-024-07627-3

**Published:** 2024-07-05

**Authors:** Guozheng Jiang, Luchun Xu, Yukun Ma, Jianbin Guan, Ningning Feng, Ziye Qiu, Shibo Zhou, Wenhao Li, Yongdong Yang, Yi Qu, He Zhao, Zeyu Li, Xing Yu

**Affiliations:** 1 Department of Dongzhimen Hospital, Beijing University of Traditional Chinese Medicine, Beijing, 100700 China; 2 Department of Honghui-Hospital, Xi’an Jiaotong University, Xi’an, 710054 China

**Keywords:** Lumbar degenerative disease, Sacral screw, Screw loosening, Bicortical fixation, Torque

## Abstract

**Background:**

Sacral screw loosening is a typical complication after internal fixation surgery through the vertebral arch system. Bicortical fixation can successfully prevent screw loosening, and how improving the rate of bicortical fixation is a challenging clinical investigation.

**Objective:**

To investigate the feasibility of improving the double corticality of sacral screws and the optimal fixation depth to achieve double cortical fixation by combining the torque measurement method with bare hands.

**Methods:**

Ninety-seven cases of posterior lumbar internal fixation with pedicle root system were included in this study. Based on the tactile feedback of the surgeon indicating the expected penetration of the screw into the contralateral cortex of the sacrum, the screws were further rotated by 180°, 360°, or 720°, categorized into the bicortical 180° group, bicortical 360° group, and bicortical 720° group, respectively. Intraoperatively, the torque during screw insertion was recorded. Postoperatively, the rate of double-cortex engagement was evaluated at 7 days, and screw loosening was assessed at 1 year follow-up.

**Results:**

The bicortical rates of the 180° group, 360° group, and 720° group were 66.13%, 91.18% and 93.75%, respectively. There were statistically significant differences between the 180° group and both the 360° and 720° groups (*P* < 0.05). However, there was no statistically significant difference between the 360° group and the 720° group (*P* > 0.05).The rates of loosening of sacral screws in the 180° group, 360° group, and 720° group were 20.97%, 7.35% and 7.81%, respectively. There were statistically significant differences between the 180° group and both the 360° and 720° groups (*P* < 0.05). However, there was no statistically significant difference between the 360° group and the 720° group (*P* > 0.05). The bicortical 360° group achieved a relatively satisfactory rate of dual cortical purchase while maintaining a lower rate of screw loosening.

**Conclusion:**

Manual insertion of sacral screws with the assistance of a torque measurement device can achieve a relatively satisfactory dual cortical purchase rate while reducing patient hospitalization costs.

## Introduction

The pedicle screw internal fixation system has always played a crucial role in the treatment of degenerative diseases of the lumbar spine due to its three-dimensional fixation structure and favorable biomechanical properties [1]. However, loosening of the implanted screws due to various reasons has become one of the main complications during postoperative follow-up, which not only affects the fusion effect but also increases the risk of further surgery for patients [2–4]. Due to the unique anatomical structure and position, the sacrum has a significantly higher incidence of screw loosening after placement compared to other segments fixed with pedicle screws [5, 6].

Scholars both domestically and abroad have been devoted to exploring methods to improve the fixation strength of sacral screws and reduce the incidence of screw loosening. These methods include: dual or triple cortical fixation of the sacrum, dual sacral screw fixation, adjunct iliac screw fixation, and sacral screw fixation with bone cement [7–9]. Human cadaveric studies and finite element analysis have shown that dual cortical fixation can significantly increase the torque and pull-out strength of screws, enhancing the mechanical stability within the system [8, 10]. Performing dual cortical fixation of the sacrum is not only highly feasible but also within the expertise of seasoned spinal surgeons, who can attain partial dual cortical fixation relying on tactile feedback during screw insertion. This proficiency is particularly enhanced with the aid of three-dimensional image navigation and intelligent technologies [11, 12], which subsequently enhance the likelihood of screws reaching the optimal dual cortical zone.Therefore, this method is currently recognized by the majority of physicians.

However, the additional three-dimensional navigation equipment limits the widespread clinical use of this technology. Moreover, solely relying on tactile feedback to achieve dual cortical fixation of the sacrum requires high demands on clinical doctors and makes it difficult to stably and accurately control the depth of screw insertion. Therefore, how to achieve sacral dual cortical fixation safely, stably, and conveniently in clinical practice remains a focus of research.

Measuring the torque changes during screw insertion can quantify the tactile sensation, assisting the operator in determining whether the screw has achieved dual cortical fixation.So this study investigates whether combining tactile sensation with torque measurement can enhance the success rate of sacral screw dual cortical fixation, building upon the foundation of manual screw insertion technique.

## Information and methods

### General information

Inclusion Criteria: 1. clearly diagnosed as lumbar degenerative disease (lumbar disc herniation, lumbar spinal stenosis) based on clinical symptoms and imaging data, ineffective after conservative treatment for more than six months, or effective after conservative treatment for more than three months but with recurrent episodes that seriously interfere with the individual's daily life and normal work.2. The lumbosacral segments retain some movement.3. Dual-energy X-ray absorptiometry (DXA) measurement of T-score ≤ -2.5 SD [13]. (All patients were assessed for T-scores based on lumbar spine (L1-L4) bone density using the Horizon W ® DXA system (Hologic, Inc)).

Exclusion criteria: 1. lumbar isthmic cleft synostosis, structural retroflexion, severe narrowing of the intervertebral space; 2. Severe osteoporosis, imbalance in the coronal plane of the spine, lateral displacement of the vertebral body > 1 cm, compression of the vertebral body wedge change in the coronal plane of the vertebral body > 1/3, severe stenosis of the intervertebral foramina, accompanied by severe menopausal symptoms, psychiatric disorders, and chemical substance dependence.

**Fig. 1:**
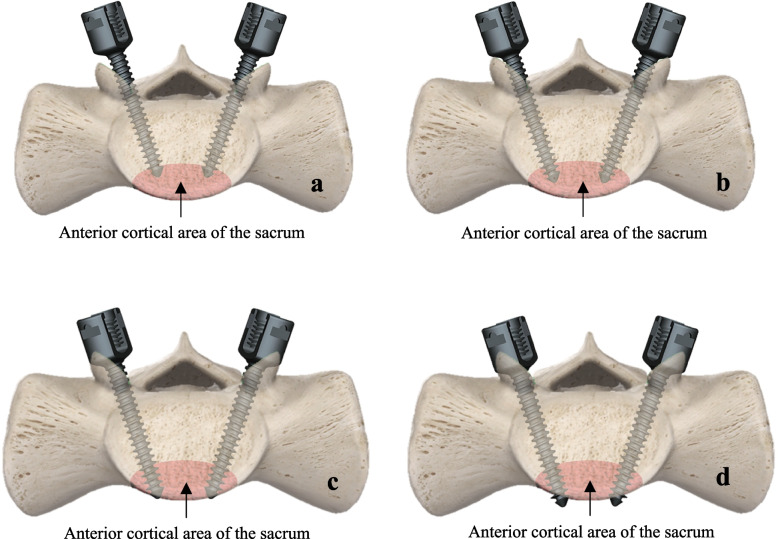
**a** The sacral screw has just reached the contralateral cortical area of the sacrum. **b** The sacral screw, after reaching the contralateral cortical area of the sacrum, is further rotated by 180°. **c** The sacral screw, upon reaching the contralateral cortical area of the sacrum, is further rotated by 360°. **d **The sacral screw, after reaching the contralateral cortical area of the sacrum, is further rotated by 720°

A total of 97 patients who underwent lumbar decompression with dynamic rod fixation for treatment from September 1, 2019 to September 1, 2021 were included. These patients were randomly divided into three groups using a random number table. All patients experienced preoperative lower back pain, radiating pain in the lower limbs, and/or intermittent claudication, and had not responded to conservative treatment.All of the above cases included sacral 1 segments and the torque values were measured intraoperatively using a digital torque meter (Item No.: RSX-30NLT Origin: Taiwan). When the screw is expected to reach the contralateral cortical area of the sacrum (the surgeon feels an increased resistance while turning the screw, and there is a noticeable need to apply greater wrist rotational force to continue screwing in the screw), it is further rotated by 180°, resulting in the double cortical 180° group, where one thread is inserted into the vertebral body. Alternatively, when the screw is rotated by 360°, it forms the double cortical 360° group, with two threads inserted into the vertebral body. Furthermore, when the screw is rotated by 720°, it constitutes the double cortical 720° group, with three to four threads inserted into the vertebral body. Refer to Fig. 1 for illustration.

The study was carried out in compliance with the ethics committee of Dongzhimen hospital affiliated to Beijing university of Chinese medicine, and informed consent was obtained from all subjects and/or their legal guardian(s). Additionally, the study was carried out in accordance with the moral guidelines that were established by the Declaration of Helsinki and its following amendments.

### Surgical methods

**Fig. 2:**
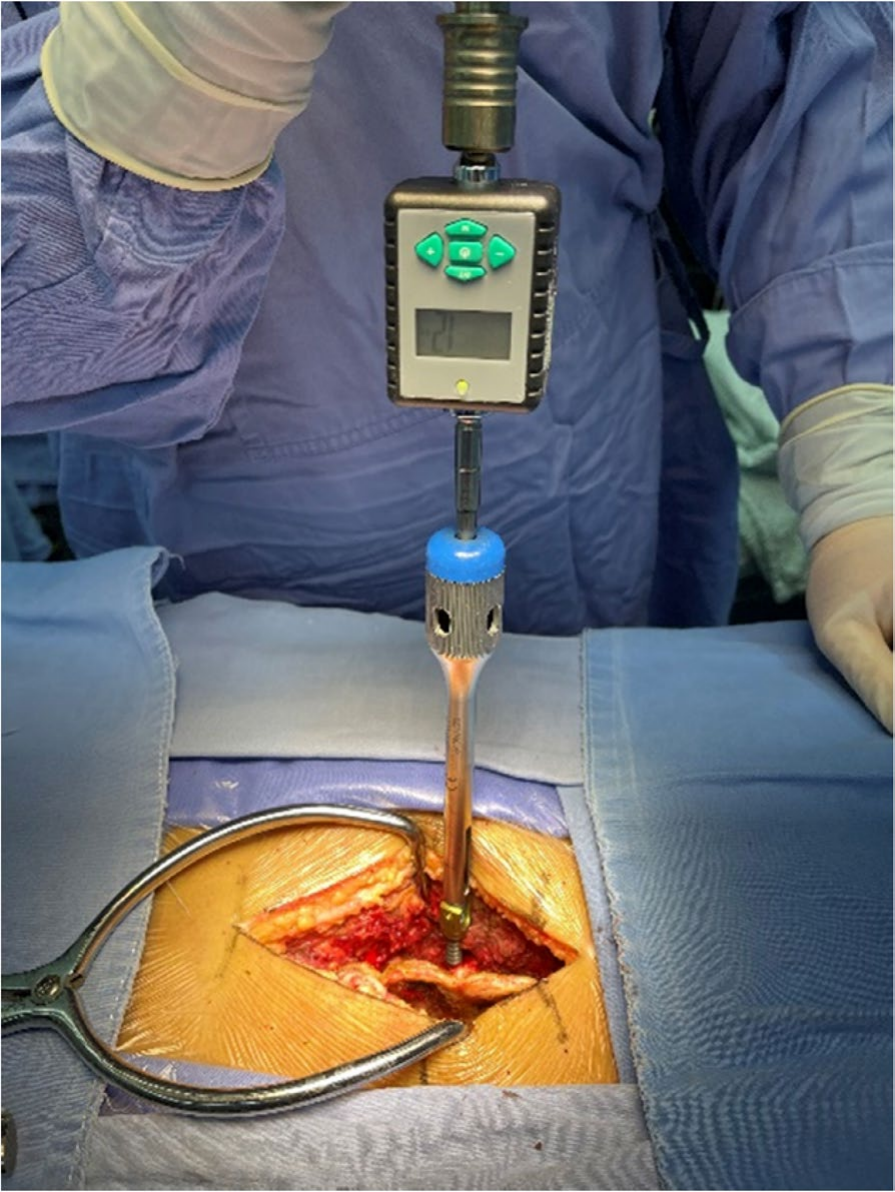
Intraoperative measurement of screw torque using digital torque gauge during nail placement process

All patients were placed in the prone position under general anesthesia. The surgeon approached the lumbosacral spine through a midline incision, sequentially dissecting the skin, subcutaneous tissue, and lumbodorsal fascia. The sacrospinal muscles were detached bilaterally, exposing the spinous processes, laminae, and lateral aspects of the facet joints at the operative levels while ensuring the protection of the facet joints and joint capsules. Intraoperatively, C-arm fluoroscopy was utilized to confirm the accurate positioning of the operative levels. Subsequently, appropriately sized polyaxial pedicle screws (catalog numbers: 12VTLP45/55/62/70–25 ~ 80) were sequentially inserted. For segments L1-5, screws were oriented as parallel as possible to the superior endplates of the vertebrae. For the S1 segment, after penetrating the anterior cortex using a direct approach with the road cone (based on intraoperative changes in road-opening resistance, where resistance significantly increases, roughly judging the arrival at the contralateral cortex area, then hammering in 3—5 mm is sufficient), dual cortex screws are inserted. The surgeon feels an enhanced twisting sensation and continues screw insertion for 180°, 360°, or 720° when screw insertion resistance increases, as shown in Fig. 2. A digital torque measuring instrument is used during the screw placement process to record torque changes.Narrowing of the vertebral segments was addressed using rongeurs to remove hypertrophic spinous processes and laminae, as well as thickened ligamentum flavum, with care taken to protect the facet joints. Decompression of the lateral recess was performed using a minimally invasive technique until complete relief of neural and central canal compression was achieved, and any extruded nucleus pulposus was explored and removed. After thorough decompression of the operative segments, titanium or dynamic rods were inserted in a specific direction and secured with locking screws. Adequate hemostasis and irrigation were ensured in the surgical area, and a lumbar drain was placed extradurally. The incisions were closed layer by layer, and compression dressing was applied.

### Perioperative management

Postoperative antibiotics were routinely given to prevent infection, hemostasis, analgesia and neurotrophic treatment, and the drainage was observed and removed within 24-36 h after surgery. All patients were placed in the lying position after the operation, and wore a waist cuff to get out of bed for activities according to the actual situation, and the wearing time of the waist cuff was 1–2 months.

### Evaluation indicators

 During sacral screw placement, a torque meter was applied to record the torque values at the starting placement point, at the midpoint of screw placement, and at 180°, 360°, or 720° of rotation of the screw after it reached the contralateral cortex, and the torque of screw placement in the bicortical 720°group was continuously recorded.

 Routine preoperative X-ray, CT, and MRI examinations were performed, and lumbar CT.

Review was performed 7 days after the operation, and plain scanning was performed along the sacral screw nailing channel section to assess the effect of bicortical fixation of the screws.

 Screw loosening was determined by 2 experienced physicians at regular postoperative visits, and in case of inconsistency, it was determined by the surgeon. The diagnostic criteria for screw loosening were [14]: a translucent band of ≥ 1 mm on both sides of the screw in the lumbar spine in the frontal and lateral X-rays at the last follow-up visit. All cases were followed up for ≥ 24 months at least [15]. 

### Statistical analysis

All data were analyzed statistically using SPSS 22.0 software. Continuous data were represented as mean ± standard deviation. The t-test was utilized for inter-group comparisons when the data demonstrated normal distribution and homogeneity of variance. Alternatively, in cases of non-normal distribution or heterogeneous variance, the Kruskal–Wallis H rank sum test was employed. Inter-group comparisons of categorical data were conducted using Pearson's chi-square test, with statistical significance denoted by *P* < 0.05.

## Results

### General

A total of 97 patients were included in the three groups, 46 males and 51 females, aged 45–76 years (58.75 ± 10.24 years), and a total of 194 sacral screws were measured. There were 31 patients in the bicortical 180° group, 34 patients in the 360° group, and 32 patients in the 720° group. The average follow-up time of all cases was 27 months (24–33 months). There was no statistically significant difference in age, gender, mobility of the lumbosacral regions and follow-up time among the three groups of patients, *P* > 0.05, as shown in Table 1.

**Table 1:** General information of the three groups of patients

clusters	Number of patients	Age (years)	Sex (m/f)	mobility of the lumbosacral regions(°)	Follow-up time (months)
Bicortical180° group	31	57.93 ± 9.78	15/16	8.53 ± 0.73	26 ± 7
Bicortical360° group	34	59.41 ± 10.24	18/16	8.67 ± 0.49	27 ± 6
Bicortical720° group	32	58.56 ± 8.96	17/15	8.45 ± 0.81	25 ± 7

### Bicortical rate of sacral screws

In this investigation, sacral screws breaking through the anterior sacral cortex were regarded as achieving bicortical fixation, and the screws achieving bicortical fixation in the three groups of patients are shown in Table 2. The difference between the bicortical rate of sacral screws in the bicortical 180° group and that in the bicortical 360° group and the 720° group was statistically significant, with *P* < 0.05; there was no statistically significant difference between the bicortical rate of sacral screws in the bicortical 360° group and that in the 720° group, with *P* > 0.05.

**Table 2:** Bicortical rates and differences in sacral screws

clusters	Number of double cortical screws (pieces)	bicorticality(%)	*P*-value	
			Bicortical 360° group	°Bicortical 720°group
Bicortical 180° group	41	66.13%	< 0.001	< 0.001
Bicortical 360° group	62	91.18%	/	0.577
Bicortical 720° group	60	93.75%	0.577	/

### Loosening of sacral screws

The difference between the sacral screw loosening rate of the bicortical 180° group and that of the bicortical 360° and 720° groups at the last follow-up was statistically significant, *P* < 0.001; there was no statistically significant difference between the sacral screw loosening quality rate of the bicortical 360° group and that of the 720° group, *P* > 0.05. See Table 3.

**Table 3:** Sacral screw loosening rates and differences

clusters	Number of loose screws (pieces)	Looseness(%)	*P*-value	
			Bicortical 360° group	°Bicortical 720°group
Bicortical 180° group	13	20.97%	0.025	0.035
Bicortical 360° group	5	7.35%	/	0.921
Bicortical 720° group	5	7.81%	0.921	/

### Depth of sacral screw placement in relation to torque

**Fig. 3:**
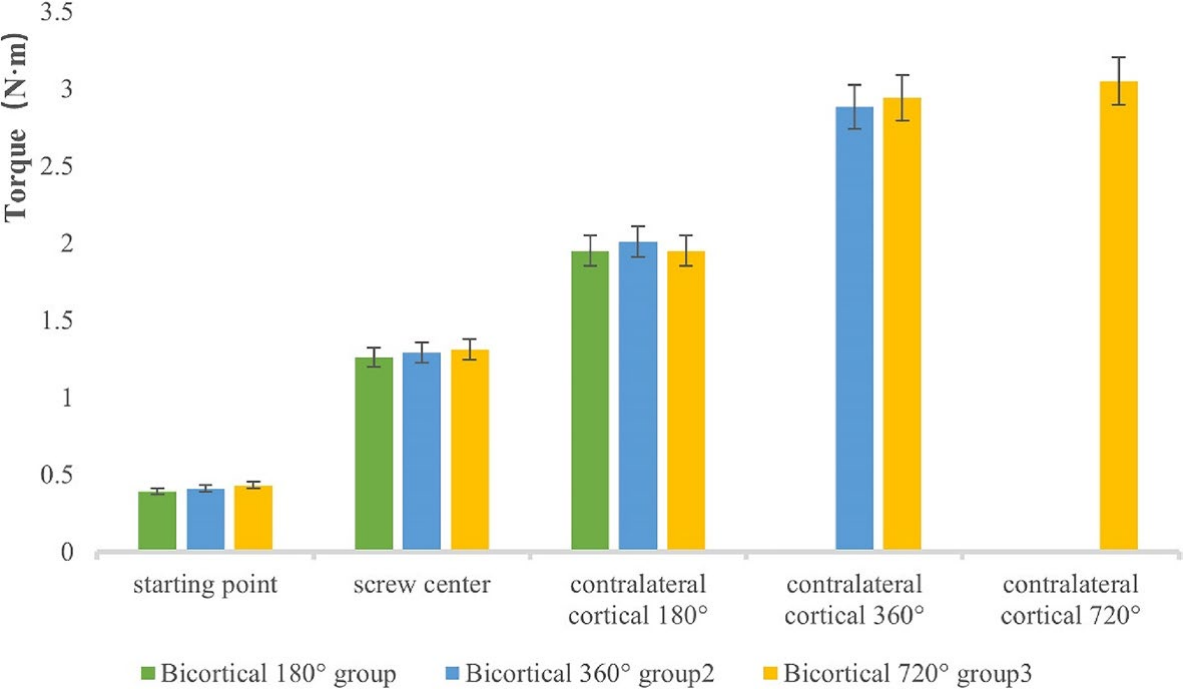
Depth and mean torque graph of sacral bone screw placement

**Fig. 4:**
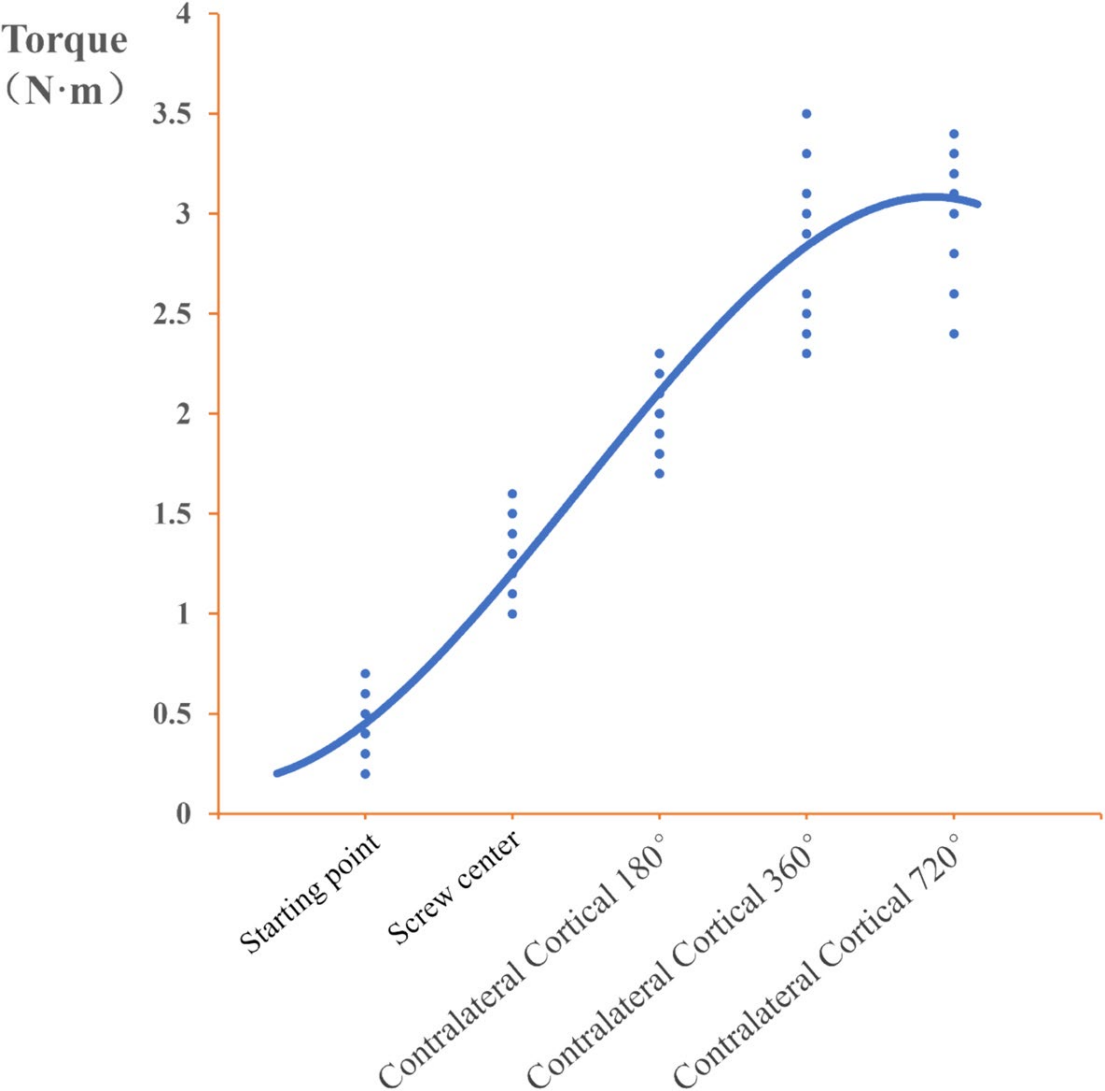
Trend graph of sacral bone screw placement depth and torque variation (The graph was created using the least squares method for curve fitting)

The average torque values of the screws in the 180°, 360°, and 720° bicortical groups were basically around 0.4 N·m during initial placement; the torque values increased gradually when the screws were placed at the midpoint; the torque values increased significantly when the screws reached the contralateral cortex and continued to be screwed into the 180° group compared with the midpoint of the screws; the torque values of the screws continued to be inserted into the 360° group increased significantly compared with that of the screws inserted into the 180° group, with a *P* < 0.05; and the torque values of the screws continued to be inserted into the 720° group significantly increased compared with that of the screws inserted into the 180° group, with a *P* < 0.001. Torque values increased slightly when the screw was continued to 720° compared with 360°, *P* > 0.05.Changes in torque during sacral screw placement in each group are shown in Table 4 and Fig. 3. Trends in depth of sacral screw placement versus torque are plotted in Fig. 4.

**Table 4:** Table of changes in sacral screw torque^①^

clusters	starting point	screw center	180°	360°	720°
Bicortical 180° group	0.39 ± 0.12	1.26 ± 0.17	1.95 ± 0.21	/	/
Bicortical 360° group	0.41 ± 0.15	1.29 ± 0.22	2.01 ± 0.29	2.88 ± 0.37	/
Bicortical 720° group	0.43 ± 0.14	1.31 ± 0.16	1.95 ± 0.19	2.94 ± 0.35	3.05 ± 0.27

Note: ①Torque unit is N·m

### Subgroup analysis of screws at different depths

Due to varying bone densities among patients, the tactile resistance felt when inserting screws into the sacrum at the same depth can differ. Therefore, a subgroup analysis of screws at different depths was conducted, as shown in Table 5.

**Table 5:** Subgroup analysis of screws at different depths

Screw depth (mm)	Number of patients	Bicortical 180° group	Bicortical 360° group	Bicortical 720° group	Torque (N·m)
30 ~ 34 group	33	30	3	0	2.03 ± 0.19
35 ~ 39 group	36	0	33	3	2.76 ± 0.23
40 ~ 45 group	28	0	2	26	3.03 ± 0.31

Among patients with screw depths between 30 and 34 mm, 90.9% (30/33) were in the bicortical 180° group, with a maximum torque value of 2.03 ± 0.19 N·m, showing no statistically significant difference compared to the bicortical 180° group (*P* > 0.05). Among patients with screw depths between 35 and 39 mm, 91.7% (33/36) were in the bicortical 360° group, with a maximum torque value of 2.76 ± 0.23 N·m, showing no statistically significant difference compared to the bicortical 360° group (*P* > 0.05). Among patients with screw depths between 40 and 45 mm, 92.9% (26/28) were in the bicortical 360° group, with a maximum torque value of 3.03 ± 0.31 N·m, showing no statistically significant difference compared to the bicortical 720° group (*P* > 0.05).

### Typical cases

**Fig. 5:**
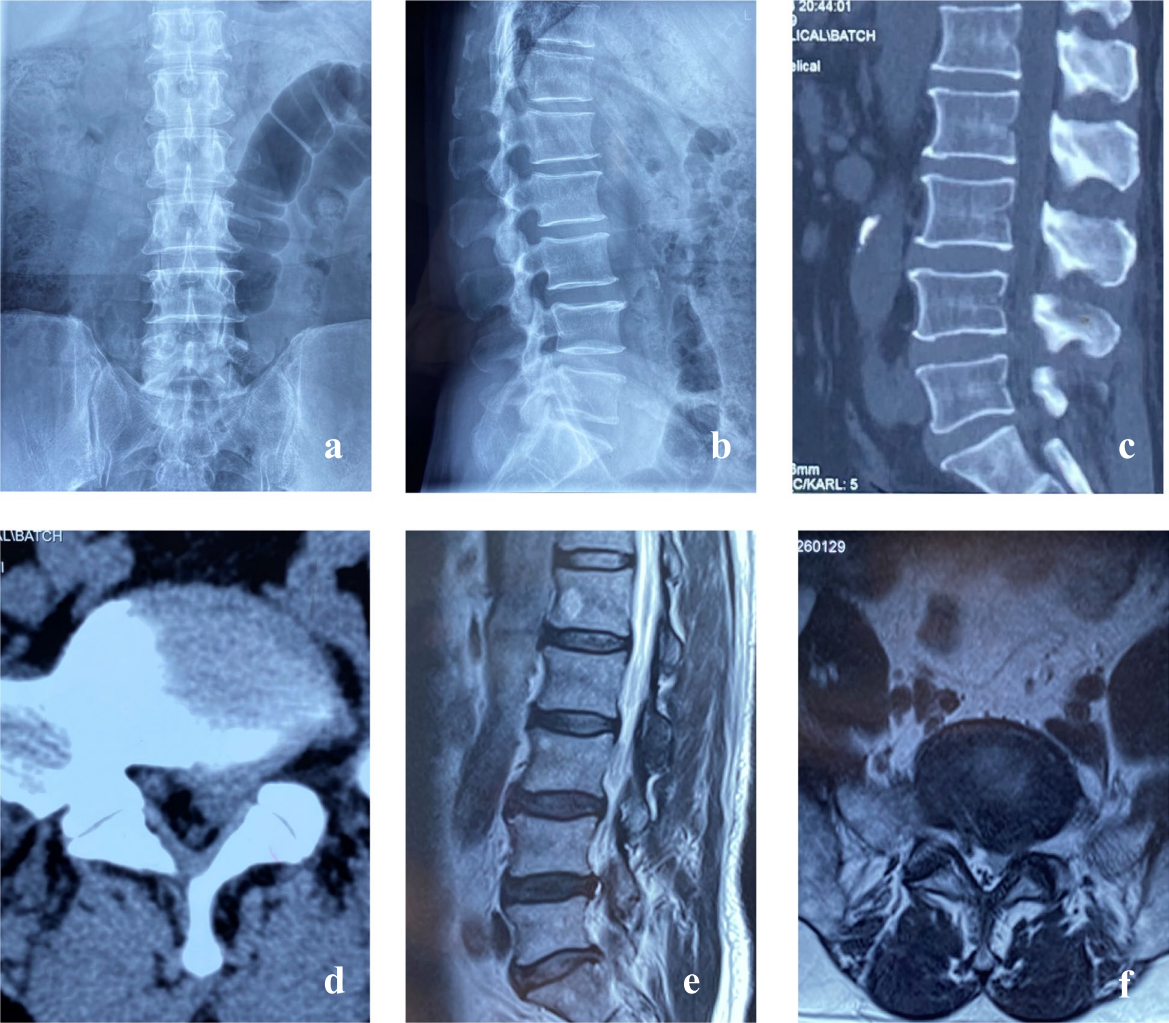
Preoperative anteroposterior and lateral X-ray images (**a**, **b**) reveal multiple lumbar degenerative changes, with a narrowed L5-S1 intervertebral space. Preoperative CT scans (**c**, **d**) and MRI scans (**e**, **f**) indicate a pronounced stenosis of the L5-S1 spinal canal, accompanied by protrusion of the nucleus pulposus

**Fig. 6:**
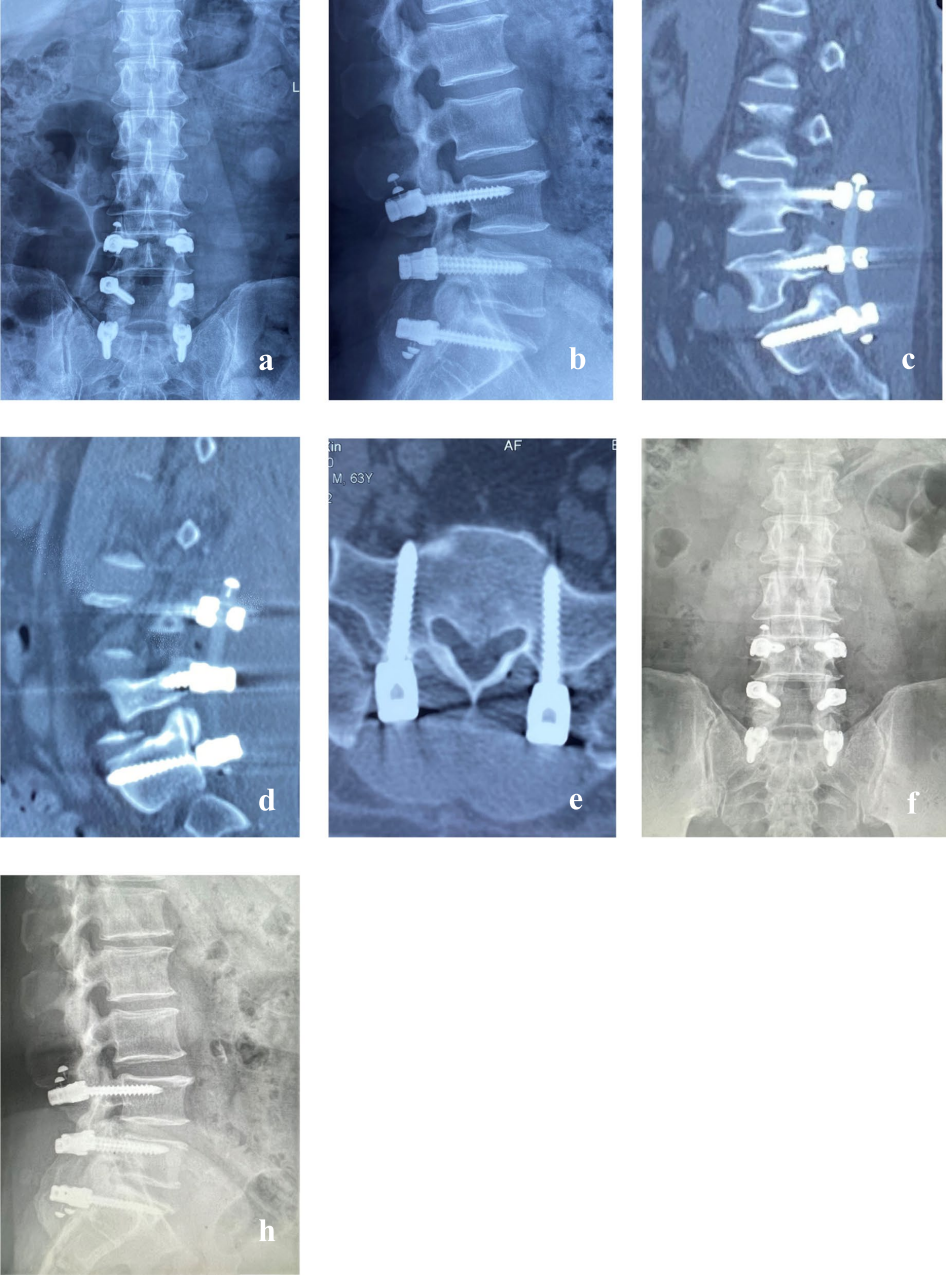
Postoperative Day 7, anteroposterior and lateral X-ray images (**a**, **b**) showed satisfactory screw fixation and appropriate depth of sacral screw placement. Postoperative Day 7, lumbar spine CT scans (**c**, **d**, **e**) revealed bilateral sacral screws achieving bicortical fixation. Postoperative Month 28, anteroposterior and lateral X-ray images (**f**, **h**) demonstrated well-maintained positions of bilateral sacral screws, with no evidence of loosening or fracture

Bicortical 360° group case: 63-year-old male patient with "back pain for 30 years, with left lower limb pain, numbness, and intermittent claudication for 1 month", admitted to hospital, clear diagnosis of lumbar spinal stenosis, posterior lumbar laminectomy decompression with transpedicular system internal fixation, pain symptoms significantly improved 1 week after surgery. The patient walked 3000 m without experiencing any intermittent claudication at the most recent checkup.The pre- and post-operative imaging data are shown in Fig. 5 and Fig. 6.

## Discussion

To achieve bicortical fixation of the sacral screws, we used a combination of freehand and torque measurements. The results showed that rotational resistance was not apparent at the initial stage of screw insertion, and the initial torque values of the three groups of sacral screws were similar, essentially in the range of 0.35–0.45N·m; as the screws rotated to about the midpoint of the screws, the operator's perceived resistance gradually increased, which was essentially the same as the torque values, and the torque values were maintained in the range of 1.20–1.35N·m. As the depth of screw insertion increases and it enters the cortical area on the contralateral side, the surgeon distinctly notices a rise in rotational resistance. This is accompanied by a notable increase in the observed torque value compared to the previous readings. Following the screw's breakthrough into the contralateral cortical area of the sacrum and achieving a 360-degree rotation, there is a consistent rise in torque values. However, as the rotation continues beyond 360 degrees up to 720 degrees, the tactile rotational resistance remains unchanged basically. Although there is an increase in torque values during this phase, the differences are not substantial. We believe that this may have occurred because, as shown in Fig. 1, the bicortical 360° group of screws had essentially broken through the contralateral cortex of the sacrum, and the bicortical 720° group of screws had partially penetrated the contralateral cortex of the sacrum compared to the bicortical 360° group of screws. Although more deeply penetrated than the bicortical 360° group of screws, the length of the screws within the sacrum was essentially the same. In addition, the screws used in this study were isothreaded universal screws. If the length of the vertebral body's grip on the screw is the same, the grip is also basically the same.

The increase in resistance encountered during sacral screw insertion is, to some extent, dependent on bone quality. This study conducted a subgroup analysis of screws at different depths, showing that the trend of screw depth changes is generally consistent with changes in torque values and tactile resistance. The reason for this consistency lies in the inclusion of patients with osteoporosis, with no statistical differences among the three groups. Additionally, patients with significant sclerotic hyperplasia of the sacrum were excluded from this study because satisfactory grip strength can be achieved by placing screws in the sclerotic areas of these patients, resulting in higher torque values and a lower incidence of postoperative screw loosening, making dual cortical fixation unnecessary. Furthermore, to avoid injury to the L5 nerve and iliac vessels, the screw insertion angle was maintained between 20° and 30° relative to the sagittal plane. This ensured the consistency of the criteria for achieving dual cortical fixation in each patient.

Bicortical fixation of sacral screws can effectively reduce the incidence of screw loosening. Based on a comprehensive follow-up period spanning a minimum of 24 months, the observed screw loosening rate within the bicortical 180° group closely aligned with figures reported in the existing literature, registering at 20.97% [16]. Remarkably, within the bicortical 360° and 720° groups, patients experienced comparatively fewer instances of sacral screw loosening, yielding corresponding rates of 7.35% and 7.81%. Noteworthy is that these rates within both these groups either matched or, in certain instances, even fell below the documented screw loosening rates observed in prior investigations [17]. The data above indicate that after a noticeable increase in manual resistance during the sacral screw insertion process, continuing to screw in up to 360°, no need to 720°, generally achieves satisfactory screw stability, resulting in a lower loosening rate of the screws.

All patients enrolled in this study had osteoporosis, a condition associated with a relatively high incidence of sacral screw loosening. Consequently, a strategy of dual cortical fixation was pursued. Patients with evident sacral sclerosis on preoperative CT scans were excluded from the study. This exclusion was based on the understanding that satisfactory screw purchase could be achieved by placing screws in the sclerotic area. This approach resulted in higher screw torque and lower rates of postoperative screw loosening, obviating the need for deliberate pursuit of dual cortical fixation.The patient population included in this study consists of individuals with retained mobility in the lumbar spine. The selection of an internal fixation device with a dynamic rod aims to preserve a certain degree of mobility while ensuring lumbar spine stability, thereby mitigating postoperative stiffness and reducing the incidence of adjacent segment disease (ASD) [18, 19]. Dynamic rods allow for a certain degree of mobility in the lumbosacral region, and research has shown that using dynamic rods for posterior spinal fixation can promote bone growth around the screws, thereby increasing screw stability [20, 21].

Nevertheless, screw loosening remains a common complication of dynamic pedicle screw fixation systems and is one of the key factors leading to surgical failure. Studies have shown that the percentage of screw loosening is approximately 1%-27% in patients with normal bone [2, 17, 22] and up to 60% in patients with osteoporosis [23, 24]. Additionally, the L5-S1 segment, serving as the transitional zone between the lumbar spine and the sacrum, bears a significant portion of the body's weight. The shear forces at the lumbosacral angle further exacerbate local loading, necessitating stronger grip strength to maintain stability at the bone-screw interface following screw insertion [25].

It should be noted that the distribution of cancellous bone density within the sacrum is non-uniform. It is primarily characterized by a significant density of cancellous bone adjacent to the anterior cortical area, resulting in correspondingly elevated CT values. Conversely, in the posterior cortical region of the sacral wings on both sides, there is a lower density of cancellous bone, leading to relatively lower CT values [5]. The characteristics of the changes in feel and torque during sacral screw placement in this study are also consistent with this bone distribution characteristic of the sacrum. Previous studies have shown that there is little difference in the average CT values between the vertebral bodies of the lumbar spine and the sacrum [5, 26]. However, the trabecular bone distribution within the vertebral bodies of the lumbar spine is relatively uniform, while the density distribution of trabecular bone within the sacrum is uneven. This may result in less gripping force of screws in the sacrum compared to the lumbar spine, consequently leading to a less secure fixation compared to the lumbar spine.In general, lumbar pedicle screws can be placed into 2/3 of the vertebral body to obtain a satisfactory holding force [27], while sacral screws are often placed into the same depth can not meet the requirements.The lumbosacral joint has a relatively higher degree of mobility, and postoperatively, there is a higher rate of loosening of sacral screws compared to other lumbar segments, especially in patients with osteoporosis [28]. Hence, it is crucial to emphasize the immediate gripping force and medium-term stability of the screws, particularly in these cases. In order to solve the above problems, scholars in various fields have made a lot of efforts, and one of the important concepts is to improve the stability after placement by making the screws contact as much cortical bone as possible. Research has shown that bone density increases as one moves closer to the cortical region within the pedicle. Cortical bone screws offer better grip strength compared to conventional pedicle screws [29]. The diameter of the pedicles at the S1 level is usually larger than that of the screws, resulting in less grip strength of sacral screws compared to normal pedicle screws.Therefore, sacral bicortical fixation is an effective way for improving the stability of the internal fixation system after screws implantation.

Bicortical fixation of sacral screws can effectively increase the stability of the screws, which has been verified by in vitro studies and finite element analysis. Smith [30] conducted a torque study on 25 human specimens for single-cortical and bicortical screw insertion and found that the torque value for bicortical screw insertion was higher than that for single-cortical screws, and the difference between the two values was even more pronounced in the younger specimens. Zhu et al. [31] conducted a study using 11 specimens of young male sacra to investigate the torque during the insertion of both single and double cortical screws, as well as the pullout strength after subjecting the screws to 20,000 cycles of loading following their placement. The findings demonstrated a noteworthy increase in both the insertion torque and pullout strength for double cortical screws when compared to single cortical screws. Eltes [32], Fradet [8] et al. confirmed by finite element analysis that bicortical fixation of the sacrum can significantly increase the strength of sacral screws fixation compared with that of single-cortical fixation of the sacrum.

The spine surgeon is challenged to obtain safe, precise, and rapid bicortical fixation of the sacrum. We are presently capable of accurately placing screws within the optimal location of the bicortex by utilizing advanced 3D image-guided navigation and intelligent procedural techniques. However, it's important to note that this approach does result in an elevation of both time and cost for the patient undergoing the procedure [33]. Experienced spinal surgeons are able to achieve partial bicortical fixation based on tactile feedback. The thread pitch selected for this study is uniform. During the screw insertion process, the trabecular bone density of the sacrum passed through at the initial stage is smaller, while it increases as it approaches the contralateral cortex. The tactile resistance feedback experienced by the surgeon during screw insertion shows a gradual and uniform increase at the initial stage, and a significant increase in resistance when reaching the contralateral cortex region. Experienced surgeons will determine whether to continue screw insertion and the depth of screw insertion based on the strength of tactile resistance [34].

This study employs torque measurements to quantitatively analyze the variations in tactile perception experienced by surgeons during the screws placement procedure. The primary objective is to enhance control over nail insertion depth and thereby elevate the overall safety of the procedure. The study findings reveal a gradual increase in torque as the screw traverses the cortical bone of the sacral vertebral arch and enters the vertebral body. A distinct surge in torque becomes evident as the screw approaches the contralateral cortex. Upon breaching this cortex, the torque registers an almost 100% surge compared to its level during the midway insertion of the screw. Surgeons are able to discern a palpable rise in resistance as they continue to advance the nail. As the screw's rotation progresses, the torque rises steadily until it exceeds 360 degrees of inward rotation, beyond which no significant further increase in torque is observed.

This study pioneers the integration of torque applied during screw insertion with the surgeon's tactile feedback, thereby enhancing the success rate of sacral cortical fixation while concurrently mitigating the expenses associated with utilizing three-dimensional image navigation. Moreover, in comparison to three-dimensional intelligent navigation systems, the torque measurement device exhibits a shorter learning curve and does not appreciably prolong the surgical duration.

## Conclusion

This study suggests that compared to three-dimensional navigation systems, manual insertion of sacral screws with the assistance of a torque measurement device can achieve a relatively satisfactory dual cortical purchase rate while reducing patient hospitalization costs. However, larger-scale and longer-term studies are still needed to determine its long-term safety and effectiveness.

## Limitations

Firstly, all cases in this study were from the same single-center surgical team, and the selection of sacral screw depth to some extent depended on the clinical experience of the operators, which introduces a certain degree of subjectivity. Additionally, the follow-up time in this study was relatively short, and continuous attention and further research are needed for the mid-to-long-term loosening of the screws. Lastly, the relatively small sample size included in this study raises the possibility of selection bias, which may result in errors in the findings.

## Data Availability

The datasets used and/or analysed during the current study available from the corresponding author on reasonable request.
